# Kinematic Analysis of Plasticization and Transportation System of Tri-Screw Dynamic Extruder

**DOI:** 10.3390/polym16233252

**Published:** 2024-11-22

**Authors:** Bin Xue, Jun Li, Qu Yang, Guiting Wu, Danxiang Wei, Yijie Ding, Zhenbin Du, Mingshi Huang

**Affiliations:** 1 School of Mechanical and Automotive Engineering, Guangxi University of Science and Technology, Liuzhou 545616, China; lee1996640@gmail.com (J.L.); wuguiting0122@163.com (G.W.); weidanxian@gmail.com (D.W.); 15390108655@163.com (Y.D.); 15280716953@163.com (Z.D.); 17677452673@163.com (M.H.); 2 School of Mechanical and Marine Engineering, Beibu Gulf University, Qinzhou 535011, China

**Keywords:** tri-screw extruder, vibration force field, kinematics, shear rate, elongational rate

## Abstract

With the growing demand for high-performance polymer composites, conventional single- and twin-screw extruders often fall short of meeting industrial requirements for effective mixing and compounding. This research investigates the kinematic behavior of the plasticization and transport mechanisms in tri-screw extruders when subjected to a vibrational force field. The study specifically examines how applying vibrational force technology can improve the efficiency of polymer mixing. Vibration force field means that in a three-screw mechanism, an axial vibration is applied to the middle screw to produce a vibration force field. Through the development of mathematical and physical models, this study analyzed the motion dynamics of the screw and the influence of a vibrational force field on polymer transport and mixing efficiency. The findings indicate that, in comparison to traditional twin-screw extruders, tri-screw systems can achieve higher shear and elongational rates, leading to enhanced polymer mixing uniformity. Furthermore, applying an axial vibrational force field significantly influenced the shear and elongational strain rates of the material, thereby optimizing its rheological behavior and processing quality. This research not only establishes a theoretical foundation for the design and optimization of tri-screw extruders but also opens new pathways for the efficient processing of high-viscosity composite materials.

## 1. Introduction

New materials promote the progress of human society. Since the 20th century, polymer materials have become essential in modern society due to their unique structures, ease of processing, and cost effectiveness [1]. Advances in science, technology, and economic development have expanded their applications across diverse fields, including defense, electronics, healthcare, transportation, and environmental protection [2,3,4,5,6,7,8]. This growing importance has driven increased national focus on both the theoretical and applied research of polymer materials. Extrusion remains the most common processing method for polymers, extensively used in industries such as plastics, rubber, food production, and pharmaceuticals [9,10]. In this process, extruders continuously move the plastic melt from the feed zone to the discharge zone using the rotational motion of screws. The kinematic behavior of screw extruders plays a pivotal role in ensuring the stability of the extrusion process and the quality of the final products. Thus, studying the kinematic principles of screw extruders is vital for enhancing both process stability and product quality.

Traditional single-screw extruders are the most widely used extrusion equipment. Studies by Guerrero [11] and Mikulionok [12,13] have examined models of single-screw extruders, focusing on the effects of screw geometry and friction coefficients within the feed zone. Carrot [14] explored the solid conveying and mixing performance of twin-screw extruders. While twin-screw extruders offer enhanced mixing capabilities compared to single-screw systems, they still face challenges like low fill rates and decreased production efficiency, particularly when processing materials such as glass fibers, plant fibers, and biomass [14,15,16,17,18]. In a single-screw extruder, material transport relies on friction between the material, the screw, and the barrel. However, if the material’s viscosity changes or the screw speed fluctuates, conveying can become unstable. For example, low-viscosity materials may experience slippage, leading to inconsistent output. In a twin-screw extruder, the intermeshing of two screws creates a forced conveying effect, which ensures more stable forward movement of the material. This design offers greater adaptability to materials with varying viscosities. Even at high speeds, twin-screw extruders maintain stable conveyance, minimizing issues related to changes in material properties.

In recent years, the rising demand for high-performance polymer composites has outpaced the mixing capabilities of conventional single-screw and twin-screw extruders, driving the need for the development of more advanced extrusion equipment. Zhu, Yang [19,20] found through simulation that three screws have better working efficiency and mixing effect than single screws and twin screws. A three-screw extruder introduces an additional screw to the traditional twin-screw design, creating a more complex and diverse flow field. This unique flow structure subjects materials to multidirectional shearing, stretching, and mixing during extrusion. For instance, when producing high-performance blends, the three-screw extruder facilitates finer dispersion and mixing of different polymer phases, resulting in a more uniform microstructure and enhanced material properties—capabilities that are challenging for single- and twin-screw extruders to achieve. Some scholars have also carried out a lot of research in theory [21,22,23,24]. Zhu [25] and Wang [26] studied the flow and mixing mechanism of three-screw extruder by using computational fluid dynamics and Carreau–Bird fluid model. The results showed that the three-screw extruder had better mixing efficiency than the traditional twin-screw extruder.

Academician Qu Jinping introduced the vibration force field into single-screw extruders, pioneering a new approach to polymer blending and modification, with notable success. Through visual enhancements to the screw extrusion system, the study explored the impact of the vibration force field on the melting and plasticizing processes during material conveyance. A new extrusion and melting mechanism was developed to explain the effects of this technology [27,28]. Feng [29] and colleagues studied the impact of vibration force fields on the mixing capacity of polymer extruders, developing an analytical model of pulsating total shear strain and analyzing the dynamic laminar mixing process. Their findings showed that the application of vibration increased the total shear strain in the melt within the spiral channel. Specifically, higher vibration frequencies and amplitudes led to greater shear stress experienced by the melt during transport. For example, increasing the vibration frequency from 3 Hz to 7 Hz and the amplitude from 1 mm to 3 mm resulted in a 50% increase in total shear strain. The results demonstrated that introducing a vibration force field enhanced both shear strain and extrusion stability [30,31,32,33].

**Figure 1:**
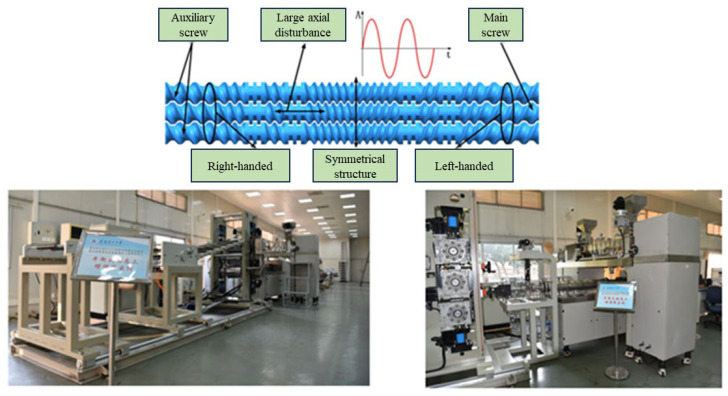
Three-screw dynamic extruder.

Under the guidance of advisor Professor He Hezi, in the present research, the vibratory field was applied to a triple-screw extruder, leading to the design and development of a balanced triple-screw dynamic extruder, as depicted in Figure 1. The extruder’s symmetrical screw structure effectively counteracts the axial resistance generated during material plasticization and feeding, reducing the axial dynamic load on the end bearings of the screws. This design also facilitates the generation of large-amplitude axial vibrations in the central main screw. Cai [34] studied the effect of introducing vibration on the mixing of three-screw extruder PP/mEPDM, and the results showed that when the fixed amplitude was 0.5 mm (or the frequency was 15 Hz), the impact strength of the sample increased first and then decreased with the increase of the vibration frequency (or amplitude) when the mEPDM content was 10%, 15%, and 20%. When the frequency was equal to 15 Hz and the amplitude equal to 0.5 mm, the impact strength reached its maximum value. For the sample with mEPDM content of 25%, the impact strength increased approximately linearly with the increase of the vibration frequency and amplitude within the experimental parameters. When the frequency was equal to 20 Hz and the amplitude equal to 0.6 mm, the impact strength reached its maximum value of 54.4 kJ/m^2^, which was about 9% higher than that without introducing vibration and 18 times that of the pure PP steady-state impact strength. Zhao [35] studied the mixing effect of a vibratory field on EPDM/PP blends, and the results showed that EPDM/PP blends prepared by a three-screw device with a vibratory field (axial vibration frequency of 20 Hz, amplitude of 0.20 mm) had better performance indicators than those prepared by a conventional three-screw device without a vibratory field. Compared with the samples prepared by a twin-screw extruder, the samples prepared by the vibrating three-screw device showed an increase of 22.7% in tensile strength, an increase of 20% in elongation at break, and an increase of 54.2% in tear strength.

In order to study the mixing effects of the vibratory field introduced to this new triple-screw extruder, this paper conducts a dynamic theoretical analysis of the plasticization transport system of the triple-screw dynamic extruder.

## 2. Screw Physical Model

**Figure 2:**
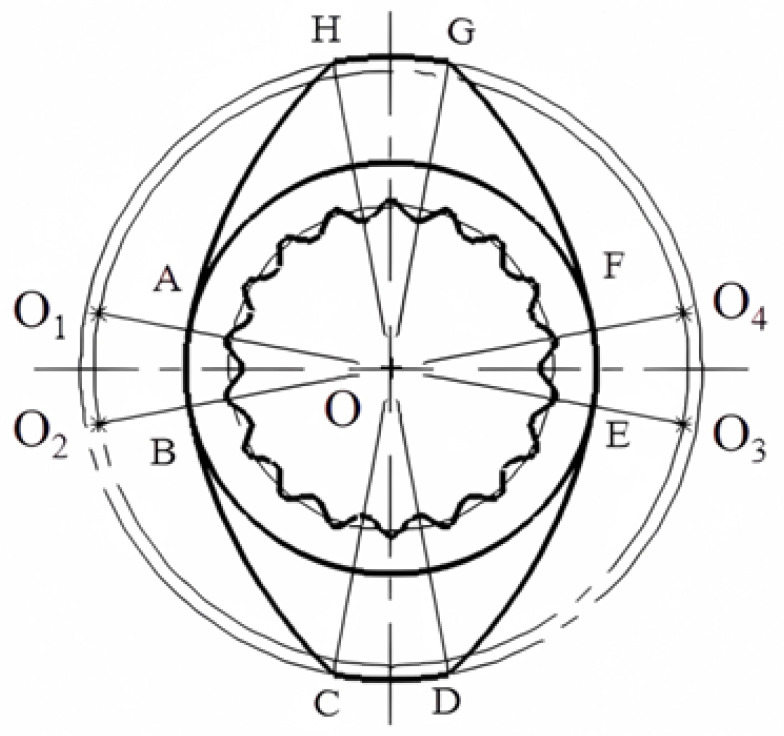
Cross-sectional profile of screw.

To develop the physical model of the extruder screw, the basic structure of the screw was first analyzed. The extrusion system of the dynamic three-screw extruder features three screws, each with an identical double-headed thread. The cross-sectional profile of each screw, as viewed perpendicular to the screw axis, is illustrated in Figure 2. The whole outline consists of eight arcs: *AB*, *BC*, *CD*, *DE*, *EF*, *FG*, *GH*, and *HA*, where the centers of the four arcs *AB*, *CD*, *EF*, and *GH* are *O* points. The radii of *AB* and *EF* are $r_{b}$, and the radii of *CD* and GH are $r_{s}$. The centers of *DE*, *FG*, *HA*, and *BC* are at points *O*_1_, *O*_2_, *O*_3_, and *O*_4_, respectively, and the radius of each arc is equal to the center distance between adjacent screws.

**Figure 3:**
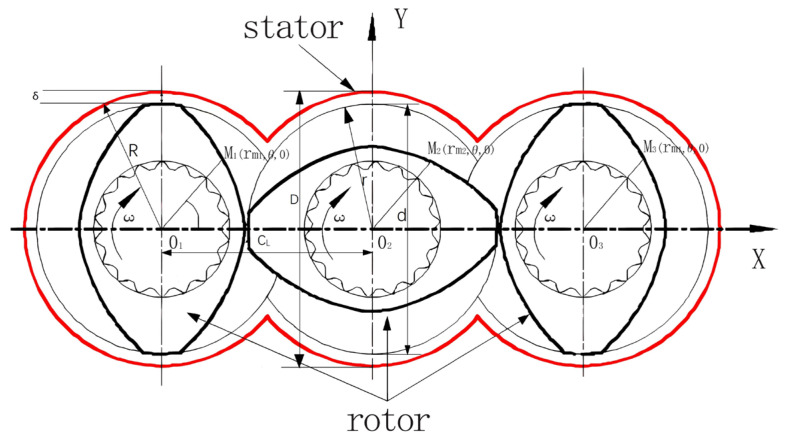
Establishment of coordinate system of three-screw plasticizing conveying system and marking of main geometric dimensions.

The plasticizing and transportation system of the balanced three-screw dynamic extruder consists primarily of a barrel and three screws arranged within the barrel’s inner cavity. The spiral threads of all three screws are aligned in the same direction, with the screws initially installed in a lined configuration. The left and right screws are installed with identical phases, offset by 90 degrees relative to the phase of the central screw. The cross-sectional physical model of the plasticizing and transportation system for each screw is shown in Figure 3. The key geometric dimensions of the components within the conveying system are as follows:

1 The diameter of the inner hole of the barrel is *D* and the radius is *R*;
2 The diameter of the cross-section circle of the three screws is *d*, and the radius is *r*;
3 The center distance of the screw is *C_L_*;
4 The clearance between the inner wall of the barrel and the screw is a fixed value, δ.

Based on the operating principle of the three-screw plasticizing and transportation system, a physical model was developed, with the geometric motion relationship of the system expressed through quantitative formulas. As shown in Figure 3, both rectangular and cylindrical coordinate systems are defined. The rectangular coordinate system (*X*, *Y*, *Z*) is centered at *O*_2_, the center of the inner hole of the middle screw. Additionally, three cylindrical coordinate systems, namely (*r_m_*_1_, *θ*, *Z*), (*r_m_*_2_, *θ*, *Z*), and (*r_m_*_3_, *θ*, *Z*), were established with the centers of the inner holes of the three screws, i.e., *O*_1_, *O*_2_, and *O*_3_, serving as their respective origins. The three screws rotate at the same speed, denoted as n, with identical angular velocities. In Figure 3, point *O*_1_ represents the center of the inner hole of the left barrel, serving as the origin for the cylindrical coordinate system (*r_m_*_1_, *θ*, *Z*). Point O_2_ is the center of the inner hole of the right barrel and serves as the origin for both the cylindrical coordinate system (*r_m_*_2_, *θ*, *Z*) and the rectangular coordinate system (*X*, *Y*, *Z*).

## 3. Screw Mathematical Model

### 3.1. Geometric Relationship of Screw

**Figure 4:**
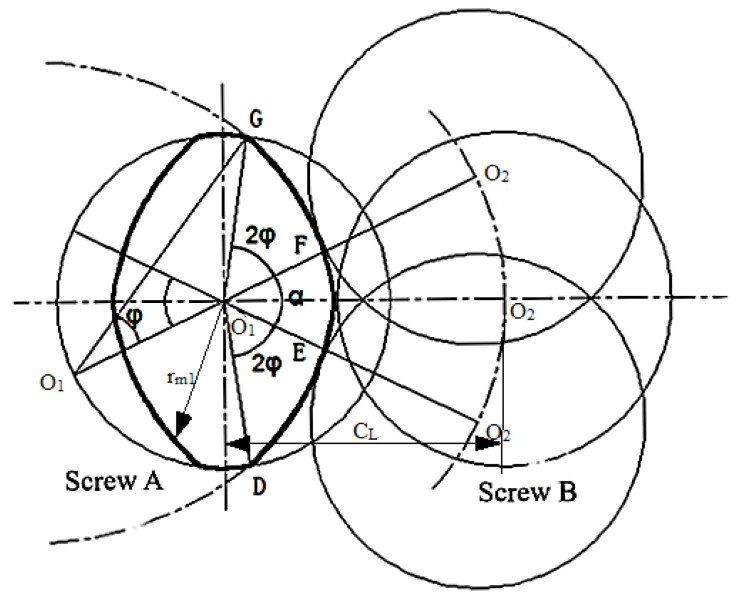
Geometric relationship of screw size.

The three-screw plasticizing and conveying system consists of three screws arranged in-line, with the middle screw acting as the symmetry axis. The arrangement of the screws on either side is identical, so the geometric relationship can be determined by analyzing the positions of the two outer screws. Consider the left two screws for analysis. First, fix screw A in place. In space, screw B will trace an arc around screw A, with the center-to-center distance, CL, as the radius. By connecting points O_1_F and O_1_G and extending this line to the arc, the two limit positions of screw B can be identified, as shown in Figure 4.

The points on the four arcs *OF BC*, *DE*, *FG*, and *GA* are shown in Figure 4. α is the included angle between the two extreme positions *OF* and *OE*, introducing angle φ in $\Delta GO_{1}O$, $OO_{1}=OG=r_{s}$, and $O_{1}G=C_{L}$ (the length of $C_{L}$ is the center distance between two adjacent screws, that is, the length between *O*_1_ and *O*_2_), from the cosine theorem:

(1) $$\varphi =\cos^{-1}(C_{L}/2r_{s})=\cos^{-1}(\rho_{c}/2)$$

**Figure 5:**
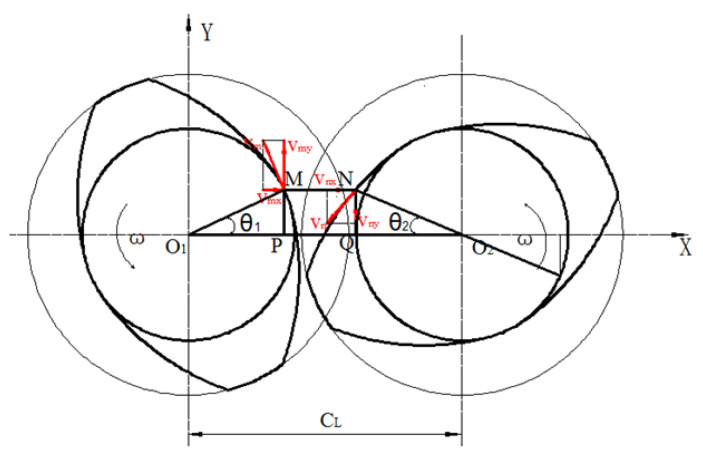
Position relationship of M point.

For the groove depth of any point *M*, the length of *MN* in the Figure 5 is the depth of spiral groove, according to Booy [36,37]. The calculation formula of groove depth is given:

(2) $$h(\theta )=r_{s}(1+\cos\theta )-\sqrt{{C_{L}}^{2}-r_{s}\sin^{2}\theta }$$

The distance from any point on the screw to the rotation center can be obtained from the obtained h(θ):

(3) $$r_{m1}=r_{s}-h(\theta )$$

$r_{s}=\frac{d}{2}$. Substituting Equation (2) into Equation (3), we can obtain the following:

(4) $$r_{m1}=\sqrt{{C_{L}}^{2}-\frac{d}{2}\sin^{2}\theta }-\frac{d}{2}\cos\theta$$

By extending Equation (4) through Newton’s binomial theorem, we can obtain the calculation equation of $r_{m1}$:

(5) $$r_{m1}=C_{L}\left[1-\frac{1}{2}{\left(\frac{d\sin\theta }{C_{L}}\right)}^{2}-\frac{1}{8}{\left(\frac{d\sin\theta }{C_{L}}\right)}^{4}-\frac{3}{48}{\left(\frac{d\sin\theta }{C_{L}}\right)}^{4}-\cdot \cdot \cdot \right]-\frac{d}{2}\cos\theta$$

The $r_{m1}$ is the distance from the center point of the screw to the outer contour of the screw, as shown in Figure 4.

Since the diameter of the screw is smaller than the center distance ($d<C_{L}$), therefore, in a certain range, the higher-order terms in Equation (5) can be ignored, leaving the following:

(6) $$r_{m1}=C_{L}-\frac{d^{2}\sin^{2}\theta }{2C_{L}}-\frac{d}{2}\cos\theta$$

In the same way,

(7) $$r_{m2}=C_{L}-\frac{d^{2}\cos^{2}\theta }{2C_{L}}-\frac{d}{2}\sin\theta$$

(8) $$r_{m3}=C_{L}-\frac{d^{2}\sin^{2}\theta }{2C_{L}}-\frac{d}{2}\cos\theta$$

When θ=2kπ (*k* = 0, 1, 2…), $r_{m1}$, $r_{m2}$, and $r_{m3}$ have a minimum value:

(9) $$r_{m1min}=r_{m3min}=C_{L}-\frac{d}{2}$$

(10) $$r_{m2mix}=C_{L}-\frac{d^{2}}{2C_{L}}$$

When θ=π+2kπ (*k* = 0, 1, 2…), $r_{m1}$, $r_{m2}$, and $r_{m3}$ have a maximum value:

(11) $$r_{m1max}=r_{m3max}=C_{L}+\frac{d}{2}$$

(12) $$r_{m2max}=C_{L}-\frac{d}{2}$$

The installation positions of the left and right screws are identical, with a phase difference of 90 degrees between the middle screw and the other two screws. The expression for the gap between the barrel and any point along the screw is as follows:

(13) $$△\delta_{m1}(\theta )=△\delta_{m3}(\theta )=R-C_{L}+\frac{d^{2}\sin^{2}\theta }{2C_{L}}+\frac{d}{2}\cos\theta$$

(14) $$△\delta_{m2}(\theta )=R-C_{L}+\frac{d^{2}\cos^{2}\theta }{2C_{L}}+\frac{d}{2}\sin\theta$$

When θ=2kπ (*k* = 0, 1, 2…), there is a maximum gap between the two screws, and the minimum gap between the two screws is as follows:

(15) $$△\delta_{m1max}(\theta )=△\delta_{m3max}(\theta )=R-C_{L}+\frac{d}{2}$$

(16) $$△\delta_{m2min}(\theta )=R-C_{L}+\frac{d^{2}}{2C_{L}}+\frac{d^{4}}{8C_{L}}$$

When θ=π+2kπ (*k* = 0, 1, 2…), the minimum clearance exists on both sides of the screw. The maximum value of the middle screw is as follows:

(17) $$△\delta_{m1max}(\theta )=△\delta_{m3max}(\theta )=R-C_{L}+\frac{d^{2}}{2C_{L}}$$

(18) $$△\delta_{m2}(\theta )=R-C_{L}+\frac{d}{2}$$

The speed at any point M on the surface of the screw 1 is $v_{m1}$, and the distance $O_{1}M$ from $M_{1}$ to $O_{1}$ is $r_{m1}$:

(19) $$v_{m1}=v_{m3}=\omega (C_{L}-\frac{d^{2}\sin^{2}\theta }{2C_{L}}-\frac{d}{2}\cos\theta )$$

Similarly, the speed $v_{m2}$ on the surface of the screw 2 is as follows:

(20) $$v_{m2}=r_{m2}\omega =\omega (C_{L}-\frac{d^{2}\cos^{2}\theta }{2C_{L}}-\frac{d}{2}\sin\theta )$$

For circular arcs *AB* and *EF*, the radius is $r_{b}$. Then, the speed of the points on the two arcs is as follows:

(21) $$v=r_{b}\omega$$

For circular arcs *CD* and *GH*, the radius is $r_{s}$. Then, the speed of the points on the two arcs is as follows:

(22) $$v=r_{s}\omega$$

### 3.2. Speed Analysis of the Screw

The screw’s outline consists of eight arcs: *AB*, *BC*, *CD*, *DE*, *EF*, *FG*, *GH*, and *HA*. The centers of the arcs *AB*, *CD*, *EF*, and *GH* are located at point *O*, resulting in a fixed gap between these points on the arc and the barrel. The centers of the arcs *BC*, *DE*, *FG*, and *HA* are located at points *O*_4_, *O*_1_, *O*_2_, and *O*_3_, respectively, and the gap between these arcs and the barrel is variable. Therefore, the shear rate of the material between the screw and the barrel can be divided into three sections, each calculated separately.

In the *AB* and *EF* sections of the arc, the shear rate of the material between the screw and the barrel is as follows:

(23) $$\overset{\cdot }{\gamma }=\frac{r_{b}\omega }{\delta_{1}}$$

In the *CD* and *GH* segments of the arc, the shear rate of the material between the screw and the barrel is as follows:

(24) $$\overset{\cdot }{\gamma }=\frac{r_{S}\omega }{\delta_{2}}$$

For the *BC*, *DE*, *FG*, and *GA* arc segments, the shear rate of materials between screw and barrel is as follows:

(25) $$\overset{\cdot }{\gamma }=\frac{\omega \left(C_{L}-\frac{d^{2}\sin^{2}\theta }{2C_{L}}-\frac{d}{2}\cos\theta \right)}{\frac{d}{2}-C_{L}+\frac{d^{2}\sin^{2}\theta }{2C_{L}}+\frac{d}{2}\cos\theta }$$

The shear rate between each point on the arc *AB* and *EF* and the drum is the minimum and is $\overset{\cdot }{\gamma }=18.3s^{-1}$. The shear rate between each point on the arc *CD* and *GH* and the drum is the maximum and is $\overset{\cdot }{\gamma }=83\;8.4s^{-1}$ The shear rate of each point of arcs *BC*, *DE*, *FG,* and *GA* can be calculated according to Equation (25). The values of each parameter in the relationship are $\omega_{L}=3\pi$ rad, $C_{L}=30$ mm, d=35.6 mm, $\delta_{2}=0.2$ mm, and ω=80 rad/s. The value range of θ is $\left[\arccos\frac{C_{L}}{2r_{s}}+2k\pi ,-\arccos\frac{C_{L}}{2r_{s}}+2\pi \left(k+1\right)\right]$ (*k* = 0, 1, 2…).

The three screws have two meshing areas, and the three screws are symmetrically distributed with the middle screw. Therefore, the left two screws were selected for analysis, and the point of the left screw θ=0° position was selected. After rotating for a certain angle, the position is at point M, and the position change is shown in Figure 5, and n is the corresponding position after rotating the right screw.

In order to calculate the relative shear speed of two screws at two points *M* and *N* in the meshing area, the distance between the two points is first determined. Since point *M* is a point on the arc *EF*, it is known that the length $r_{m1}$ of $O_{1}M$ is as follows:

(26) $$r_{m1}=r_{b}$$

The length $r_{m2}$ of $O_{2}N$ can be obtained from Equation (4) as follows:

(27) $$r_{m2}=\sqrt{{C_{L}}^{2}-\frac{d}{2}\cos^{2}\theta_{2}}-\frac{d}{2}\sin\theta_{2}$$

In $\Delta MO_{1}P$, $∠MO_{1}P=\theta_{1}$, and $O_{1}M=R_{b}$, the lengths of $O_{1}P$ and MP are as given:

(28) $$O_{1}P=r_{b}\cos\theta_{1}$$

(29) $$MP=r_{b}\sin\theta_{1}$$

In $\Delta NO_{2}Q$, $∠NO_{2}Q=\theta_{1}$, and $O_{2}N=r_{m2}$, the lengths of $O_{2}Q$ and MP are as given:

(30) $$O_{2}Q=r_{m2}\cos\theta_{2}$$

(31) $$NQ=r_{m2}\sin\theta_{2}$$

In trapezoid $MO_{1}O_{2}Q$, MN=PQ, and then, the following holds:

(32) $$MN=PQ=O_{1}O_{2}-O_{1}P-O_{2}Q$$

Substituting Equations (28) and (30) into (32) can obtain the following:

(33) $$MN=PQ=C_{L}-r_{b}\cos\theta_{1}-r_{m2}\cos\theta_{2}$$

Since $\theta_{1}$ is a known parameter, and $\theta_{2}$ is an unknown parameter, the length of MN is required, and the relationship between them needs to be established. As shown in the figure MN=PQ, the following is true:

(34) $$R_{b}\sin\theta_{1}=r_{m2}\sin\theta_{2}=\left(\sqrt{{C_{L}}^{2}-\frac{d}{2}\cos^{2}\theta_{2}}-\frac{d}{2}\sin\theta_{2}\right)\sin\theta_{2}$$

(35) $$r_{m2}=C_{L}-\frac{d^{2}\cos^{2}\theta }{2C_{L}}-\frac{d}{2}\sin\theta$$

(36) $$R_{b}\sin\theta_{1}=\left(C_{L}-\frac{d^{2}\cos^{2}\theta_{2}}{2C_{L}}-\frac{d}{2}\sin\theta_{2}\right)\sin\theta_{2}$$

**Figure 6:**
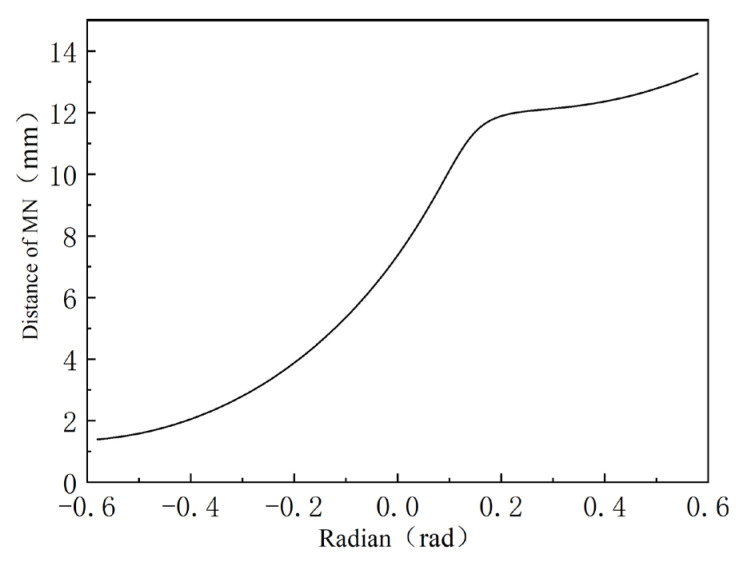
Diagram of the relationship between the clearance between two screws and rotation.

By substituting Equations (34) and (35) into Equation (36), a diagram illustrating the relationship between the clearance between two points of the two screws, labeled *MN*, is obtained, as shown in Figure 6. The figure indicates that as the rotation angle increases, the clearance between the screws also increases. Consequently, the shear rate varies as the screws rotate to different positions. The values of each parameter in the relationship are $r_{b}=11.75$ mm, $C_{L}=30$ mm, $r_{s}=17.8$ mm, and d=35.6 mm. The value range of $\theta_{1}$ is $\left[-\arccos\frac{C_{L}}{2r_{s}}+2k\pi ,\arccos\frac{C_{L}}{2r_{s}}+2k\pi \right]$ (*k* = 0, 1, 2…). The value range of $\theta_{2}$ is $\left[-\arccos\frac{C_{L}}{2r_{s}}+(2k+1)\pi ,\arccos\frac{C_{L}}{2r_{s}}+(2k+1)\pi \right]$ (*k* = 0, 1, 2…).

**Figure 7:**
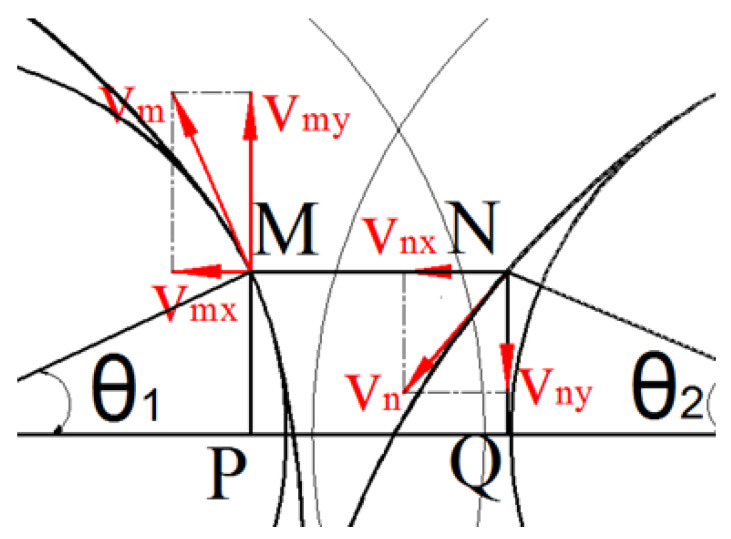
Velocity diagram between two screws.

The speed of the screw at the gap between the two screws is shown in Figure 7. According to Equations (21) and (8), the speed $v_{m}$ and $v_{n}$ at the gap between the two screws can be obtained as shown in the following formula:

(37) $$v_{m}=r_{s}\omega$$

(38) $$v_{n}=(C_{L}-\frac{d^{2}\cos^{2}\theta }{2C_{L}}-\frac{d}{2}\sin\theta )\omega$$

The material velocity in the Y-axis direction at the gap between screws shown in Figure 7 is as follows:

(39) $$v_{y}=v_{my}-v_{ny}$$

(40) $$v_{my}=v_{m}\cos\theta_{1}$$

(41) $$v_{ny}=v_{n}\cos\theta_{2}$$

Substituting Equations (37), (38), (40) and (41) into Equation (39), we obtain the following:

(42) $$v_{y}=\omega \left[r_{s}\cos\theta_{1}-(C_{L}-\frac{d^{2}\cos^{2}\theta_{2}}{2C_{L}}-\frac{d}{2}\sin\theta_{2})\cos\theta_{2}\right]$$

Therefore, the shear rate $\overset{\cdot }{\gamma }$ of the material between the two screws is as given:

(43) $$\overset{\cdot }{\gamma }=\frac{v_{y}}{MN}$$

Substituting Equations (33) and (42) into Equation (43), $\overset{\cdot }{\gamma }$ can be obtained:

(44) $$\overset{\cdot }{\gamma }=\frac{\omega \left[r_{s}\cos\theta_{1}-(C_{L}-\frac{d^{2}\cos^{2}\theta_{2}}{2C_{L}}-\frac{d}{2}\sin\theta_{2})\cos\theta_{2}\right]}{C_{L}-r_{b}\cos\theta_{1}-r_{m2}\cos\theta_{2}}$$

In the equation, the value range of q as $\theta_{1}$ is [$-arc\cos\frac{a}{2R}+2k\pi$, $arc\cos\frac{a}{2R}+2k\pi$] (*k* = 0, 1, 2…). The screw profile consists of several arcs with varying radii, each having different rotation angles. As a result, the rotation speeds of the two screws differ across these arcs in the meshing area, leading to variations in the shear rate. The specific meshing position can be analyzed in detail following the derivation process outlined above.

## 4. Analysis of the Motion Velocity of Screw by Vibration Force Field

### 4.1. Circumferential Speed of Screw

Applying an axial vibration to the intermediate screw has no effect on the circumferential speed of the screw, so the circumferential speed at a certain point on the surface of the screw when rotating is as follows:

(45) $$v_{\theta }=r_{m}\omega$$

It can be seen from the contour map of the screw that the whole contour is composed of arcs with different diameters, so the axial speed of each point on each arc is different. When the point is on *AB* and *EF* with radius $r_{b}$, its circumferential speed is as given:

(46) $$v_{\theta }=r_{b}\omega$$

When the point is on *CD* and *GH* with radius $r_{s}$, its circumferential velocity is as follows:

(47) $$v_{\theta }=r_{s}\omega$$

When the point is on the four arcs *BC*, *DE*, *FG*, and *GA*, its circumferential velocity is as follows:

(48) $$v_{\theta }=(C_{L}-\frac{d^{2}\sin^{2}\theta }{2C_{L}}-\frac{d}{2}\cos\theta )\omega$$

It can be seen from Equations (46)–(48) that the circumferential speed of each point on the screw is related to the position. When the points are on arcs *AB* and *EF*, the circumferential velocity reaches the minimum, and when the points are on arcs *CD* and *GH*, the circumferential velocity reaches the maximum.

### 4.2. Axial Speed of Screw

Because the screw is formed by scanning the cross-section along the spiral line, the screw will move in two directions during the rotation: One is in the circumferential direction, and the other is in the axial direction. Under the action of screw rotation, the material will move in two directions: One is the circumferential force exerted on the material by the circumferential rotation of the screw, and the other is the axial force exerted on the material by the axial spiral propulsion of the screw. In Section 4.1, the motion of the screw in the circumferential direction is already analyzed. Next, the motion of the screw in the axial direction is analyzed.

**Figure 8:**
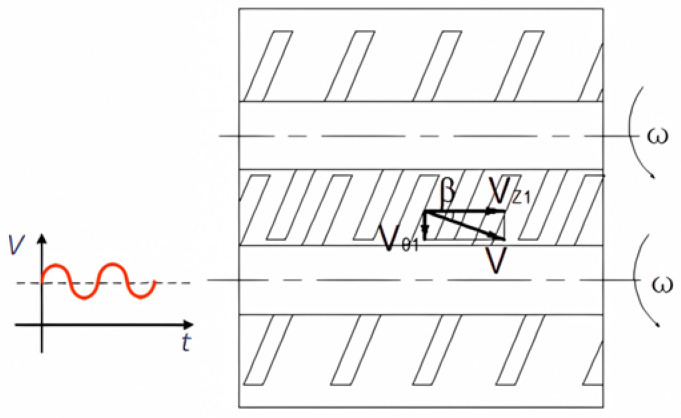
The plane expansion diagram of screw spiral section.

Next, we expand the cylindrical surfaces of the spiral sections of the two screws along the axial direction as shown in Figure 8, and the velocity of the material can be decomposed into the circumferential direction and the axial direction and recorded as $V_{\theta 1}$ and $V_{Z1}$, respectively. According to the trigonometric function theorem, the following can be obtained:

(49) $$V_{\theta 1}=V_{Z1}\tan\beta$$

(50) $$\tan\beta =\frac{T}{\pi d}$$

where β is the helix angle of the screw, T is the lead of the screw, and d is the diameter of the screw.

To enhance the mixing capabilities of the three-screw system, an axial vibration force field is applied to the middle screw. This study analyzes the impact of both axial and circumferential screw speeds on shear rate and shear plasticity. When the screw rotates uniformly in the circumferential direction while simultaneously vibrating axially, the axial vibration displacement is assumed to be as follows:

(51) $$S=A\sin\omega_{1}\;t$$

(52) $$V_{Z2}=A\omega_{1}\cos\omega_{1}\;t$$

where A is the axial vibration amplitude of the screw, and ω is the vibration circular frequency.

The introduction of axial vibration, influenced by the helix angle, also affects the circumferential direction. The relationship described by Equation (49) can be expressed as follows:

(53) $$V_{\theta 2}^{2}=V_{Z1}\tan\beta =\frac{AT\omega_{1}\cos\omega_{1}\;t}{\pi d}$$

We next superposition the velocities in the axial direction and the circumferential direction, respectively, yielding the following:

(54) $$V_{\theta }^{2}=V_{\theta 1}^{2}+V_{\theta 2}^{2}=r_{m2}\omega +V_{Z1}^{2}\tan\beta =r_{m2}\omega +\frac{AT\omega_{1}\cos\omega_{1}\;t}{\pi d}$$

(55) $$V_{Z}^{2}=V_{Z1}^{2}+V_{Z2}^{2}=r_{m}\omega \cdot \frac{\pi d}{T}+A\omega_{1}\cos\omega_{1}\;t$$

### 4.3. Screw Circumferential and Axial Deformation Rate

The meshing area exhibits the strongest mixing effect. In this section, the vibration force field is applied along the axial direction of screw 2. The analysis focuses on the trends of axial and circumferential speed changes in the meshing area between the two screws, providing theoretical support for examining the impact of the vibration force field on the mixing effect.

**Figure 9:**
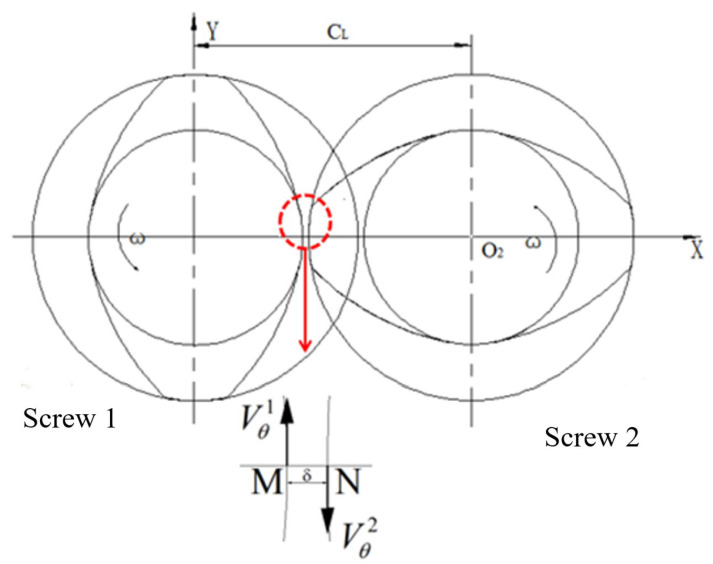
Velocity diagram between two screws.

As shown in Figure 9, the speed of any point is different at different positions on the screw profile. In order to quantitatively analyze the influence of vibration force field, we take a fixed point on the screw 1, that is, $\theta_{1}=\;2k\pi$ (*k* = 0, 1, 2…). Then, the point on the corresponding screw 1 is $\theta_{1}=\;2k\pi \;-\frac{\pi }{2}$, and the relative shear rate ${\overset{\cdot }{\gamma }}_{\theta }$ of two axial screws is as follows:

(56) $${\overset{\cdot }{\gamma }}_{\theta }=\frac{V_{\theta }^{1}-(-V_{\theta }^{2})}{\delta }=\frac{V_{\theta }^{1}+V_{\theta }^{2}}{\delta }$$

Substitute Equations (46) and (54) into Equation (56) to obtain the following:

(57) $${\overset{\cdot }{\gamma }}_{\theta }=\frac{r_{b}\omega +r_{s}\omega +\frac{AT\omega_{1}\cos\omega_{1}\;t}{\pi d}}{\delta }$$

**Figure 10:**
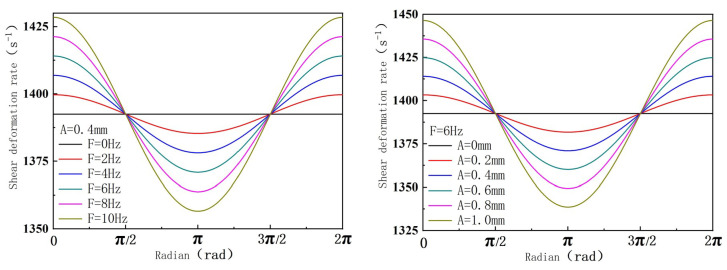
Relationship between circumferential shear deformation rate of screw surface and vibration amplitude and frequency.

Based on Formula (57), the relationship between the circumferential shear deformation rate and the screw amplitude and vibration frequency over one period is illustrated in Figure 10 (drawn by MATLAB R2021a), and the values of each parameter in the relationship are as follows: $r_{b}=11.75$ mm, $r_{s}=17.8$ mm, δ=0.2 mm, ω=3π rad/s, d=35.6 mm, and T=32 mm.

As shown in Figure 10, the circumferential shear rate increases with the vibration amplitude and frequency. This increase in shear rate aids in the dispersion of particles in the polymer matrix, enhancing the overall performance of the polymer.

In order to analyze the influence of vibration force field on shear deformation rate more intuitively, the average shear deformation rate $\bar{\overset{\cdot }{\gamma }}$ is as follows:

(58) $$\bar{\overset{\cdot }{\gamma_{\theta }}}=\frac{4\int_{k\pi }^{k\pi +\frac{\pi }{2}}\left|\left(\frac{R_{b}\omega +R_{s}\omega +\frac{AT\omega_{1}\cos\theta }{\pi d}}{\delta }\right)\right|d\theta }{2\pi }$$

**Figure 11:**
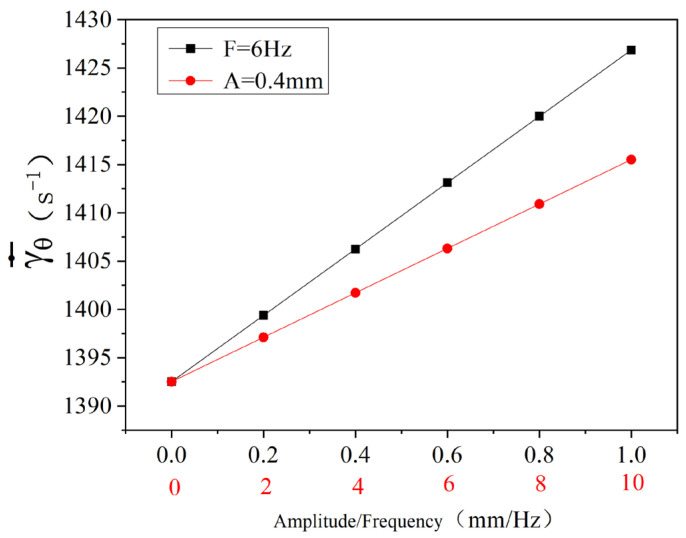
Relationship between circumferential average shear deformation rate with vibration amplitude and frequency.

According to Equation (58), the variation law of circumferential average shear deformation rate of materials with vibration and vibration frequency during one rotation of the screw is shown in Figure 11 (drawn by MATLAB R2021a). As can be seen from the figure, when the materials are mixed and conveyed in the dynamic three-screw plasticizing conveying system, the average circumferential shear deformation rate is linearly and positively correlated with the amplitude when the vibration frequency is constant (F = 6 Hz) and the amplitude changed. When the amplitude is constant (A = 0.4 mm) and the vibration frequency changed, the average circumferential shear deformation rate has a linear positive correlation with the vibration frequency. When no vibration force field is introduced (that is, the amplitude and frequency are 0), the average circumferential shear deformation rate is about 1392 $S^{-1}$. When the vibration frequency is 6 Hz and the amplitude 1 mm, the average circumferential shear deformation rate is increased to 1427 $S^{-1}$, and the increase is the maximum. This shows that the introduction of vibration force field makes the circumferential shear deformation of materials stronger.

This means that as long as one of the vibration amplitude or vibration frequency increases, or both increase at the same time, the average shear deformation rate will increase accordingly. The introduction of vibration force field makes the circumferential shear deformation of materials stronger. From the perspective of physical mechanism, the addition of vibration actually imposes additional periodic external force interference on the transportation process of materials. In the dynamic three-screw plasticizing transportation system, the rotation of the screw itself causes certain shear deformation of the material, and on this basis, the vibration force field constantly changes the relative motion state and stress of the particles in the material through its vibration amplitude and vibration frequency. The introduction of a vibration force field makes the circumferential shear deformation of materials stronger. From the perspective of physical mechanism, the addition of vibration actually imposes additional periodic external force interference on the transportation process of materials. In the dynamic three-screw plasticizing transportation system, the rotation of the screw itself causes certain shear deformation of the material, and on this basis, the vibration force field constantly changes the relative motion state and stress of the particles in the material through its vibration amplitude and vibration frequency. Macroscopically, it is reflected that the average circumferential shear deformation rate of materials is increasing, which means that the degree of shear deformation of materials per unit time is more significant, which is more conducive to the mixing and plasticizing of materials in the system; for example, it can make materials mix more evenly and have better plasticizing effect, which is of great practical significance to related industrial production processes (such as plastic processing and other fields that rely on screw plasticizing to transport materials).

The vibration force field is axially applied to the intermediate screw 2, which has the effect of stretching and compressing the material in the axial direction, so the material will be squeezed and expanded in the conveying process. The tensile deformation rate in the meshing area between the screws in the axial direction is as follows:

(59) $${\overset{\cdot }{\gamma }}_{z}=\frac{V_{Z}^{2}-V_{Z}^{1}}{l}$$

(60) $${\overset{\cdot }{\gamma }}_{z}=\frac{2\pi fA\cos\theta }{l}$$

**Figure 12:**
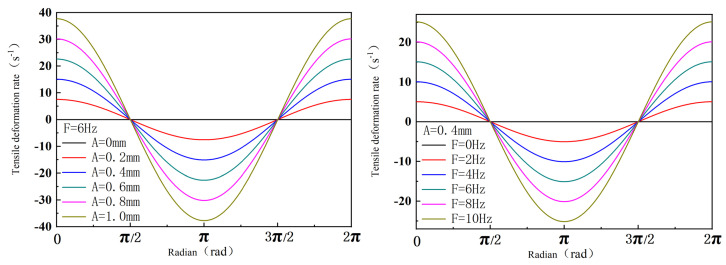
Relationship between axial tensile deformation rate of screw surface and amplitude and frequency.

According to Equation (60), the relationship between the screw amplitude and the axial deformation rate under the action of vibration frequency in a period is shown in Figure 12 (drawn by MATLAB R2021a). The figure shows that the axial shear rate increases periodically with the vibration amplitude and frequency. The axial tensile deformation rate affects the polymer differently than the shear deformation rate, leading to periodic compression–expansion of the polymer system. This improves particle dispersion in the polymer matrix, enhancing polymer performance.

Similarly, in order to analyze the influence of vibration force field on tensile deformation rate more intuitively, the average tensile deformation rate $\bar{\overset{\cdot }{\gamma_{z}}}$ is as follows:

(61) $$\bar{\overset{\cdot }{\gamma_{z}}}=\frac{4\int_{k\pi }^{k\pi +\frac{\pi }{2}}\left|\left(2\pi fA\cos\theta \right)\right|d\theta }{l}$$

**Figure 13:**
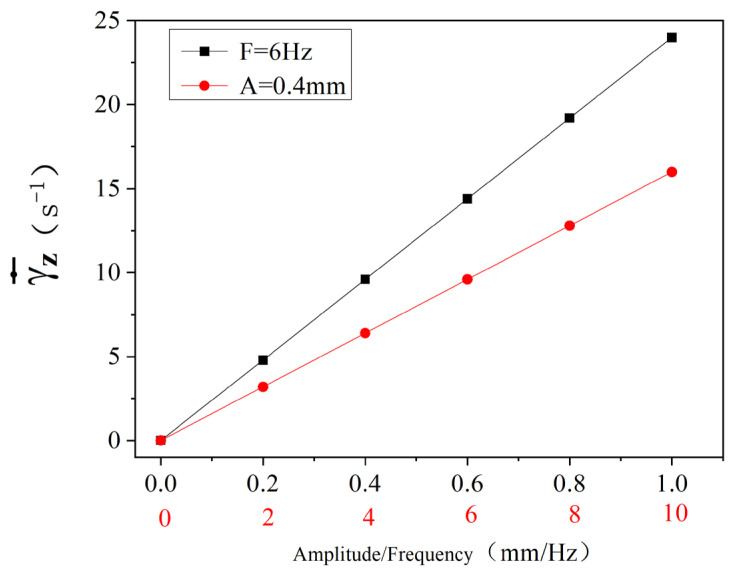
Relationship between axial tension deformation rate with vibration amplitude and frequency.

According to Equation (61), the variation law of axial average tensile deformation rate of materials with amplitude and vibration frequency during one rotation of the screw is shown in Figure 13 (drawn by MATLAB R2021a). As shown in the figure, during the mixing and transport of materials in the dynamic three-screw plasticizing system, the average axial shear deformation rate increases linearly with vibration amplitude and frequency. Throughout the screw rotation process, both the average axial shear deformation rate and its variation follow similar trends at different amplitudes and frequencies. As the amplitude and frequency increase, the average axial shear deformation rate of the materials also rises. This further indicates that the introduction of the vibration force field enhances the tensile deformation of the materials.

## 5. Conclusions

In this study, an in-depth analysis was conducted on the kinematics of the plasticizing conveying system within the balanced three-screw dynamic extruder. Mathematical and physical models were thereby established, with the aim of investigating the influence of the vibration force field on both the circumferential shear and axial tensile deformation rates. The results reveal that the introduction of the vibration force field significantly enhances the shear and tensile deformation rates of materials. Specifically, the average circumferential shear deformation rate demonstrates a linear increase with the vibration amplitude and frequency. Quantitatively speaking, the axial tensile deformation rate also presents a positive correlation with the periodicity of these parameters. This positive correlation directly contributes to the improvement of the dispersion and mixing efficiency of polymer materials. The research findings underline the efficacy of integrating axial vibration to generate a shear-tension composite flow field. This composite flow field optimizes the mixing effect and, in turn, improves the processability of polymers. These results lay a solid theoretical foundation for the design and application of advanced three-screw extruder systems, directly addressing the demand for higher efficiency in polymer composite manufacturing. By eschewing speculative applications and focusing solely on effective findings, this conclusion firmly connects the research insights with the experimental framework established in this study.

## Data Availability

Data are contained within the article.
